# Bronchial thermoplasty reduces ventilation heterogeneity measured by multiple breath nitrogen washout

**DOI:** 10.1186/s12931-020-01575-x

**Published:** 2020-11-23

**Authors:** David Langton, Kim Bennetts, Francis Thien, Virginia Plummer, Peter B. Noble

**Affiliations:** 1 Department of Thoracic Medicine, Frankston Hospital, Peninsula Health, 2 Hastings Road, Frankston, VIC 3199 Australia; 2 Faculty of Medicine, Nursing and Health Sciences, Monash University, Clayton, VIC Australia; 3 Department of Respiratory Medicine, Eastern Health, Box Hill, VIC Australia; 4 School of Human Sciences, The University of Western Australia, Crawley, WA Australia

**Keywords:** Asthma, Bronchial thermoplasty, Multiple breath nitrogen washout, Imaging

## Abstract

**Background:**

Despite demonstrated symptomatic benefit from bronchial thermoplasty (BT), the underlying pathophysiological benefits have been uncertain. The purpose of the present study was to relate clinical benefit after BT to changes in lung physiology, focusing on ventilation homogeneity assessed using multiple breath nitrogen washout (MBNW), and how this may be affected by changes in airway volume and resistance.

**Methods:**

Consecutive patients (n = 21) with severe asthma scheduled for BT, were evaluated at baseline, 6 weeks and 6 months after completion of treatment. Assessments included the Asthma Control Questionnaire (ACQ), medication usage, exacerbation frequency, spirometry, plethysmography and MBNW. Eighteen of these patients underwent detailed CT evaluation for the estimation of airway volume at baseline and then after the left lung had received BT treatment but prior to right lung treatment. Data are mean ± STDEV.

**Results:**

Patients responded to BT with an improvement in ACQ from 3.4 ± 0.8 at baseline to 2.0 ± 1.1 at 6 months (p < 0.001). Steroid requiring exacerbations fell from 3.1 ± 2.9 in the 6 months prior to BT to 1.4 ± 1.7 following BT (p < 0.001). Significant reductions in maintenance oral steroid dosing and short acting beta agonist use were observed.

Airway volume measured by CT scanning significantly increased after treatment. The FEV1 improved from 1.34 ± 0.65 l to 1.52 ± 0.76 l (p = 0.024). The Residual Volume fell from 2.87 ± 0.89 l to 2.71 ± 0.93 l (p = 0.008) and Total Airway Resistance (Raw) from 10.58 ± 6.56 to 7.64 ± 3.74 cmH_2_O.s.l^−1^ (p = 0.020). The Lung Clearance Index (LCI) was 187 ± 63% predicted at baseline and improved after treatment from 12.7 ± 3.3 to 11.8 ± 2.4 (p = 0.049). The improvement in LCI correlated with the improvement in Raw (r = 0.463, p = 0.035).

**Conclusion:**

Clinical benefit after BT is accompanied by improvements in lung physiology, including normalisation of lung homogeneity that seems to be driven by airway dilation and reduced resistance.

## Background

One of the hallmark features of severe asthma is hypertrophy of the airway smooth muscle layer [1]. Histological studies consistently demonstrate that bronchial thermoplasty induces atrophy of this airway smooth muscle layer [2, 3]. Exactly how this translates to a physiological benefit to patients is not as clearly understood. Patients do experience fewer asthmatic symptoms after BT, and fewer exacerbations [4–8]. They also have reduced reliance on medication, both short acting reliever medication and maintenance oral corticosteroids [8]. Nevertheless, improvements in spirometry are either non-existent or modest [4–8]. Mathematical modelling of the physiological behaviour of the asthmatic lung, suggests that bronchial thermoplasty should result in dilatation of the treated airways and, indeed that has been demonstrated in recent computerized tomography (CT) studies [9–11]. This modelling also suggests that the airway dilatation should improve the downstream distribution of ventilation to small airways and recent studies of ventilation homogeneity in the lung using hyperpolarized gas magnetic resonance imaging (MRI) would lend support to this proposal [12, 13].

An alternative method of measuring ventilation homogeneity is multiple breath nitrogen washout (MBNW) [14]. Compared with hyperpolarized gas MRI, MBNW has the advantages of low cost, widespread availability and repeatability on multiple occasions. Compared to CT scanning, MBNW has the advantage of the absence of ionizing radiation. MBNW has been extensively used in children with Cystic Fibrosis where it is found to be a feasible and sensitive measure of abnormal lung function [15]. In patients with asthma, MBNW indices correlate with asthma stability and airway hyper-responsiveness [16].

Therefore, in this study we sought to examine whether BT led to improvements in ventilation homogeneity as measured by MBNW, and if so whether this technique could be used to assess success after treatment with BT. Determinants of ventilation heterogeneity were also considered, specifically airway resistance assessed by plethysmography and airway volume by CT.

## Methods

### Study design

This single-centre study was conducted in an Australian university teaching hospital, experienced in providing BT. Patients were referred for BT by their treating respiratory physician if they had frequent symptoms despite optimized asthma treatment including high dose inhaled corticosteroids and two long acting bronchodilators. All patients were required to meet the European Respiratory Society/American Thoracic Society (ERS/ATS) definition of severe asthma [17]. Consecutive patients undergoing BT were enrolled.

BT procedures were conducted under general anaesthesia, and patients were electively admitted to hospital overnight following treatment. The scheduling of BT was altered so that the left lower and left upper lobes were treated in the first 2 treatment sessions. This allowed patients to be re-evaluated mid treatment with our CT scanning protocol, with the left lung completely treated but the right lung being untreated and serving as a control. This treatment protocol is reported elsewhere, but is included here in order to assist with the interpretation of the changes to the various lung function parameters [10, 18]. In this present study, patients were evaluated by spirometry, plethysmography and MBNW at baseline (in the 4 weeks prior to BT) and then again, 6 weeks and 6 months after the completion of all BT treatment.

### Assessments

The baseline clinical data recorded for each patient included age, gender, body mass index, smoking status, medication usage, exacerbation history and the Asthma Control Questionnaire 5-item score (ACQ) [19]. An exacerbation was defined by the commencement of oral corticosteroids for 3 or more days, or, in patients taking maintenance oral steroids, an increase of more than 10 mg per day from baseline for 3 days. Patient assessments were conducted by experienced clinical research nursing staff and independently of the procedural team.

Lung function measurements were made in an accredited laboratory by experienced scientific staff and to ERS/ATS standards [20]. All tests were performed in the morning, after instrument calibration, and prior to the administration of any bronchodilators. The Jaegar Masterscreen Body (Carefusion, Hoechberg, Germany) was used to perform spirometry and body plethysmography, and the predicted equations used were drawn from the Global Lung Initiative for spirometry, and from the European Coal and Steel Community for all other parameters [21, 22].

The EasyOne Pro Lab (ndd Medical Technologies, Zurich, Switzerland) was used to measure MBNW. The washout was performed using tidal breathing within a range of 0.95–1.4 L, breathing 100% oxygen until the end-tidal nitrogen concentration was consecutively below 2%. Patients were monitored during the test procedure in order to standardize the breathing manoeuvres, and to verify the absence of coughing and leaks. Two technically acceptable tests were obtained for each patient on each occasion, and the results were averaged. The Lung Clearance Index (LCI) was determined from the ratio of the Cumulated Expired Air Volume to the Functional Residual Capacity, and represents the number of gas exchanges required to clear the lung to a nitrogen concentration of less than 2% [22]. An automated phase III slope analysis of the expiratory nitrogen trace was performed during the testing to determine the convection and diffusion dependant components of ventilation inhomogeneity, termed conductive slope (Scond) and acinar slope (Sacin) respectively [22].

### Imaging studies

Non-contrast CT Scanning was performed on a 128-slice Siemens Definition AS + scanner with a helical slice thickness of 0.6 mm, rotation time of 0.6 s, detector coverage of 38.4 mm, and tube voltage of 100 kV. Two breath-hold scans were performed on each occasion—one at full inspiration, and the other at Functional Residual Capacity (FRC). All imaging was performed in a stable state, pre-bronchodilator, and prior to peri-procedural oral steroid administration. Post-acquisition, CT images were analysed independently to the investigating team by FLUIDDA (Kontich, Belgium), using Mimics (Materialise, Leuvin, Belgium), which converted the CT images into patient-specific, 3D computer models of the lung lobes and the airway dimensions [23]. The airways were partitioned into two, namely, the trachea and major bronchi which are not treated by BT, and the lobar and more distal airways down to 1-2 mm (the limits of resolution of the CT imaging), being the airways potentially treatable by BT. CT imaging was performed at baseline, and then repeated after completing BT treatment to the left lung, but prior to any BT treatment to the right lung.

### Outcomes and analysis

The primary goal of this study was to detect changes in the LCI measured 6 months after BT. Secondary outcomes related to changes in the ACQ score, spirometry and body plethysmography, and the luminal airway volume detected by CT. SPSS version 25 (IBM corporation, New York, USA) was used for all statistical analyses. Unless specified otherwise, grouped data includes all patients and is reported as mean ± standard deviation. A paired t-test (or non-parametric equivalent) was used for paired sets of data. Analysis of variance (ANOVA) was used to compare baseline data with repeated tests over time. Statistical significance was taken throughout as p < 0.05.

### Ethical considerations.

All participants provided informed consent for treatment and data collection. The project was prospectively approved by the Peninsula Health Human Research Ethics Committee.

## Results

### Baseline clinical characteristics

Twenty-four consecutive patients with severe asthma were enrolled in this study but 3 patients were withdrawn from the analysis as they were unable to successfully complete the MBNW test at baseline. The remaining 21 patients, 9 males, 12 females, had a mean age of 56.0 ± 14.3 years, and body mass index of 31.6 ± 7.0 kg/m^2^. The mean ACQ score was 3.4 ± 0.8. In the six months prior to BT, patients experienced an average of 3.1 ± 2.9 exacerbations requiring oral corticosteroids. The mean beclomethasone equivalent daily dose of inhaled corticosteroids was 1704 ± 911 ug. Fifteen patients were taking maintenance oral corticosteroids, and the mean prednisolone dose for the whole group of 21 patients was 12.5 ± 15.4 mg/day. All patients were using dual long acting bronchodilators. Patients used an average of 13.0 ± 10.4 puffs/day of short acting beta-2 agonist reliever medication. The mean peripheral blood eosinophil count was 100 ± 100 cells/ul, and the median IgE was 41 (Interquartile range 11–181) IU/ml. Twelve patients (57%) were never smokers, and there were no current smokers. The mean CT derived emphysema score was 2.2 ± 4.0% (measured at –950 Hounsfield units). The mean Forced Expiratory Volume in 1-s (FEV1) was 45.6 ± 12.9% predicted and the mean Diffusion Capacity per unit lung volume was 92.8 ± 27.8% predicted.

### Clinical Response

**Fig. 1:**
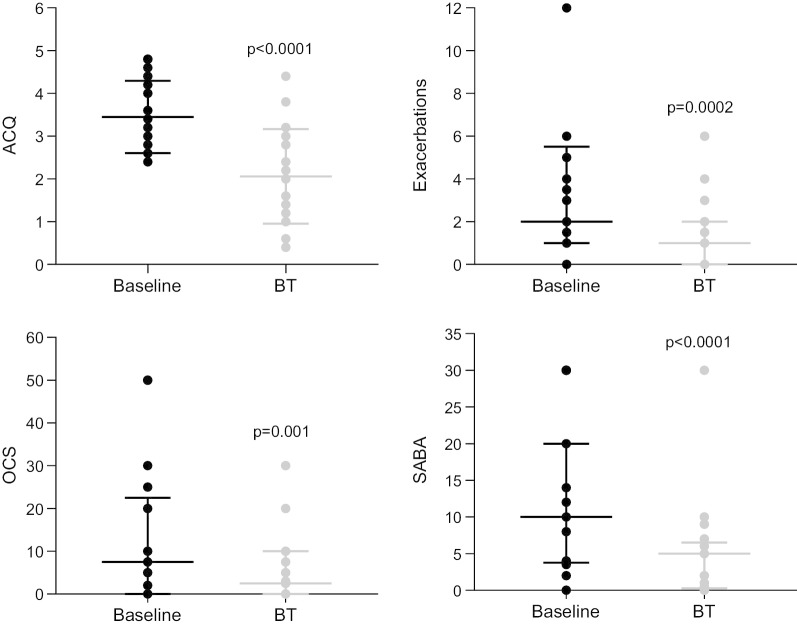
Clinical response to bronchial thermoplasty (n = 21). Asthma control questionnaire (ACQ), number of oral steroid (OCS) requiring exacerbations in the last 6 months and short acting beta agonist dose (SABA, puffs/day) were all reduced 6 months after bronchial thermoplasty. Data are mean ± standard deviation for ACQ and median [IQR] for all other measures

The response to treatment measured by clinical parameters is demonstrated in Fig. 1. The ACQ fell from 3.4 ± 0.8 at baseline, to 2.0 ± 1.2 6 weeks after BT treatment was completed, and 2.0 ± 1.1 at the 6-month reassessment (p < 0.001)—a difference of almost 3 times the minimal clinically significant change [24]. Four patients failed to achieve an improvement in ACQ of 0.5, and hence the overall group response rate measured by ACQ was 81%.

The exacerbation rate in the 6 months following completion of BT was 1.4 ± 1.7, compared to 3.1 ± 2.9 at baseline (p < 0.001). The group mean daily requirement for prednisolone fell from 12.5 ± 15.4 mg/day to 5.5 ± 7.8 mg/day (p = 0.001). The usage of short acting beta-agonist reliever treatment fell from 13.0 ± 10.4 to 5.2 ± 6.7 puffs/day (p < 0.001).

### Response to treatment: spirometry and plethysmography

The outcomes of BT measured by lung function tests are presented in Table 1. In these patients, a significant increase in prebronchodilator FEV1 was observed 6 months after BT, amounting to 170 ± 290 mls, or an increase of 12.6%. An increase in Vital Capacity (VC) was also observed, but to a smaller degree (7%). The Total Lung Capacity was unaltered by BT. Consistent with an increase in VC, a significant reduction in Residual Volume was observed. There was a substantial reduction (28%) in plethysmographically determined Total Airway Resistance after BT. The Specific Airway Resistance (sRaw) fell to a similar degree, demonstrating that the improvement in Raw did not relate to changes in lung volume.

**Table 1:** Change in Lung Function following BT

	Baseline	6 weeks post	6 months post	p
*Spirometry*				
FEV1 (l) preBD	1.34 ± 0.65	1.42 ± 0.76	1.52 ± 0.76	0.024
FEV1 (% pred)	45.6 ± 12.9	48.1 ± 14.9	52.3 ± 16.1	0.022
VC (l) preBD	2.63 ± 0.93	2.68 ± 0.93	2.82 ± 0.97	0.017
VC (% pred)	73.0 ± 13.5	76.6 ± 15.4	81.3 ± 18.9	0.029
*Plethysmography*				
TLC (l)	5.55 ± 1.33	5.66 ± 1.42	5.59 ± 1.32	0.692
TLC (% pred)	100 ± 18	103 ± 26	102 ± 22	0.652
FRC (l)	3.58 ± 1.07	3.42 ± 0.94	3.40 ± 1.05	0.218
FRC %	121 ± 38	116 ± 29	115 ± 35	0.200
RV (l)	2.87 ± 0.89	2.84 ± 1.08	2.71 ± 0.93	0.008
RV (% pred)	145 ± 41	145 ± 55	139 ± 40	0.010
Raw (cmH_2_O s l^−1^)	10.58 ± 6.56	9.09 ± 5.36	7.64 ± 3.74	0.020
Raw (% pred)	346 ± 214	297 ± 175	248 ± 121	0.019
sRaw (cmH_2_O.s)	40.8 ± 24.8	37.1 ± 25.9	30.5 ± 20.1	0.022
*Nitrogen washout*				
LCI	12.7 ± 3.3	12.2 ± 3.1	11.8 ± 2.4	0.049
Sacin (l^−1^)	0.355 ± 0.164	0.327 ± 0.179	0.299 ± 0.165	0.380
Scond (l^−1^)	0.063 ± 0.059	0.049 ± 0.062	0.069 ± 0.094	0.560

*FEV1* forced expiratory volume 1-s, *VC* vital capacity, *TLC* total lung capacity, *FRC* functional residual capacity, *RV* residual volume, *Raw* total airway resistance, *sRaw* specific airway resistance, *LCI* lung clearance index, *Sacin* acinar slope, *Scond* conductive slope

### Response to treatment: MBNW

At baseline, in this group of severely obstructed asthmatic patients, the LCI was prolonged at 12.7 ± 3.3, or 187 ± 63% of predicted values. In other words, owing to ventilation inhomogeneity, it took almost twice as long as in a healthy lung to clear the tracer gas. Following BT, a 7% improvement in LCI was observed, to 11.8 ± 2.4 (p = 0.049), indicating more homogeneous ventilation was occurring within the lung.

The phase III slope parameters were both elevated at baseline consistent with the ventilation heterogeneity demonstrated by the LCI. The Scond was 180% of the predicted value, or similar to the increase in LCI, but the mean Sacin was increased to 4 times the normal predicted value [25]. It is clear from Table 1 that there was no change in Scond following BT, but there was a trend towards improvement in Sacin. Sacin is thought to reflect diffusion dependent acinar regions of the lung. Reductions in Sacin in asthma are observed following bronchodilators, and with improved asthma control using inhaled steroids [26].

The validity of the MBNW measurements was examined by comparing the measurement of FRC made with the MBNW system, with the measurements in the plethysmograph. For 63 pairs of measurements (21 patients at 3 timepoints), the Pearson Correlation was 0.65 (p = 0.001). The FRC measurements made in the plethysmograph were higher than in the MBNW system: 3.46 ± 1.00 L by plethysmograph compared to 3.00 ± 0.88 L by MBNW (p = 0.001). This technique related difference has been previously recognized [27].

**Fig. 2:**
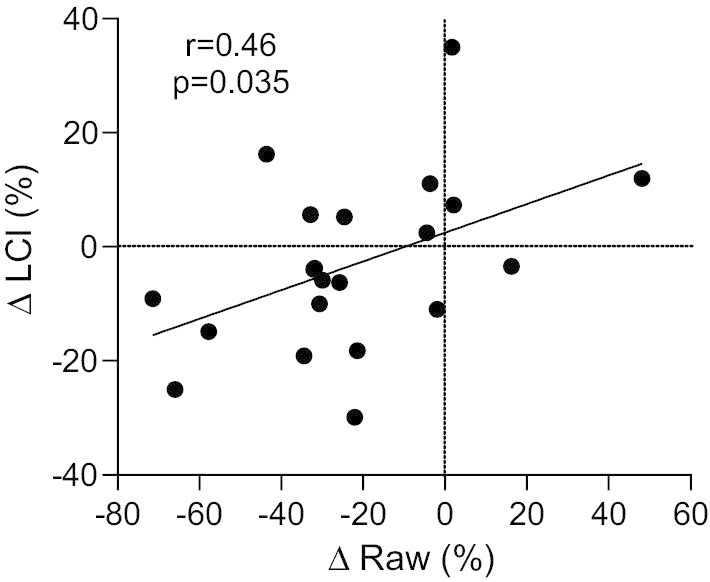
Relationship between change in total airway resistance (Raw) and change in lung clearance index (LCI) (n = 21). LCI and Raw were assessed by multiple breath nitrogen wash out and plethysmography (respectively) before and 6 months after bronchial thermoplasty. After bronchial thermoplasty, the percentage fall in resistance was positively correlated with reduced LCI

**Fig. 3:**
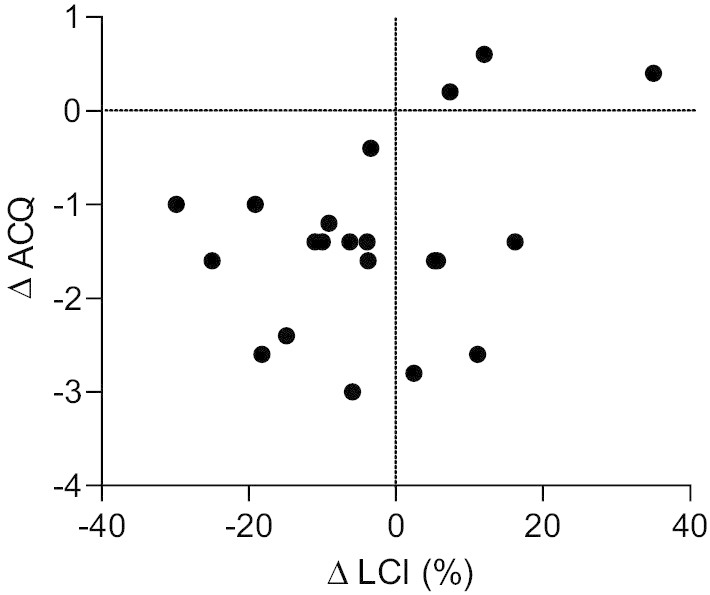
Relationship between change in lung clearance index (LCI) and change in asthma control questionnaire (ACQ) (n = 21). While the relationship between LCI and ACQ did not reach statistical significance (r = 0.41, p = 0.068), each patient who experienced an improvement (reduction) in LCI after BT also had a lower ACQ

Using Pearson correlation, the potential relationship between the changes in airway resistance (Raw) and changes in LCI, from baseline to 6 months, were examined, and a significant positive correlation was demonstrated (r = 0.46, p = 0.035) suggesting the two indices are interdependent (Fig. 2). The change in LCI was also compared with the change in ACQ (r = 0.41, p = 0.068) (Fig. 3). Although this latter correlation did not achieve statistical significance, Fig. 3 demonstrates that each patient who experienced an improvement in LCI after BT, also demonstrated an improvement in ACQ. Furthermore, the three patients in whom the ACQ deteriorated after BT, also had a deterioration in LCI.

### Response to treatment measured by CT scanning

**Fig. 4:**
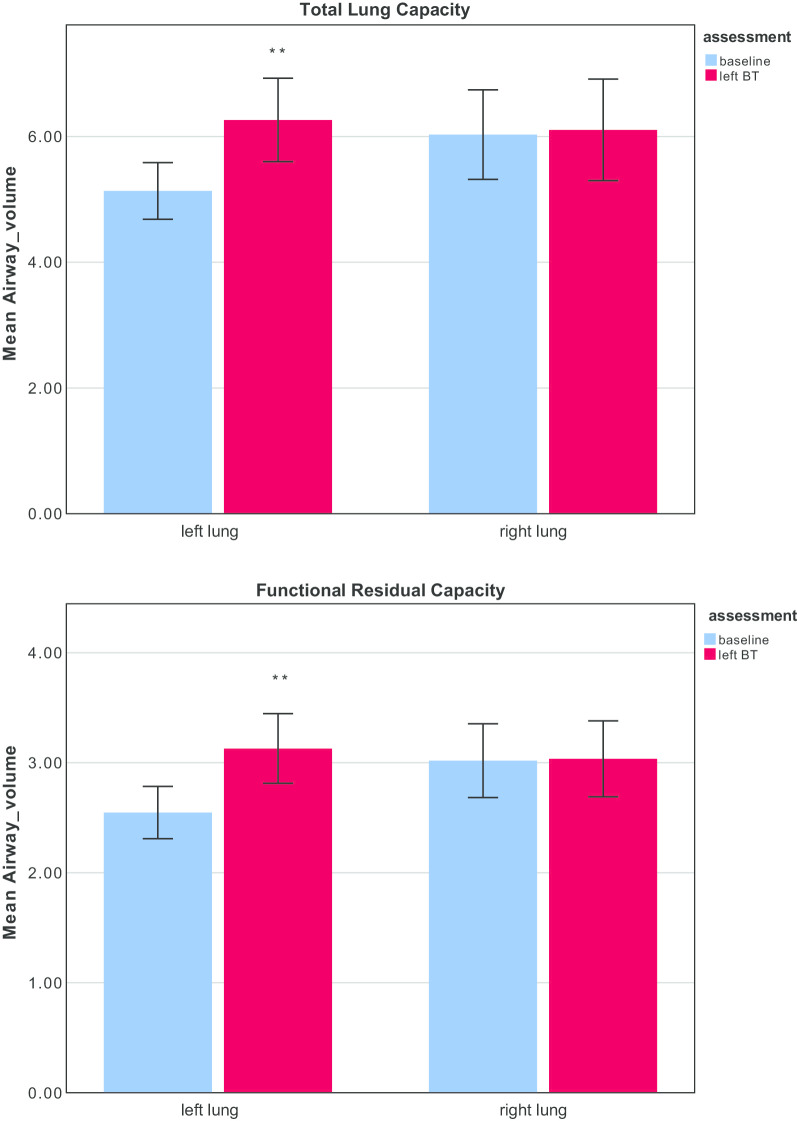
Changes in distal airway volume after bronchial thermoplasty (n = 21). Volume measurements (mL) are ‘distal to the lobar orifice’ and were increased in the treated left lung (**p < 0.01) at Total Lung Capacity (TLC, top panel) and Functional Residual Capacity (FRC, bottom panel). There were no changes in the right lung which at this time-point had not been treated by BT. Data are mean ± standard deviation

Using the described CT scanning method, the volume of air was measured in the airways distal to the lobar orifice, since these are the airways potentially treated during BT. Estimations were made both at TLC and at FRC, and for the right and left lungs. Figure 4 shows the airway volume at baseline, and then again after the left lung has been treated by BT. As expected, on the right side, no changes were observed with time. On the left side, where the airway volume is smaller at baseline owing to fewer bronchopulmonary segments, the distal airway volume increased after BT from 5.1 ± 1.9 mls to 6.3 ± 2.7 mls at TLC (p = 0.009), and from 2.5 ± 1.0 mls to 3.1 ± 1.3 at FRC (p = 0.004).

## Discussion

This group of severely affected asthmatic patients responded to BT with substantive improvements in clinical parameters such as symptom scores, exacerbation frequency and the need for medication, particularly oral corticosteroids and short acting beta agonists. Of fundamental importance, these changes were accompanied by clear improvements in lung physiology using multiple methodologies (discussed below), advancing our understanding of the mode of action of BT.

This is the first report utilising a global measurement of ventilation homogeneity to assess the effects of BT. A fall in LCI after BT is indicative of a shift to a more homogenous distribution of lung ventilation. This work is consistent with the recently published randomized trial using hyperpolarized gas MRI to identify poorly ventilated lung segments which are then specifically targeted with BT to improve symptoms in patients with severe asthma [13]. Furthermore, these real-life findings exactly conform to the predictions made by Donovan’s mathematical model of the behaviour of the asthmatic lung when subjected to BT [9].

The observed improvement in ventilation homogeneity can be explained by concomitant changes in lung physiology. There is clear dilation of airways after BT as assessed by CT, present only in lobes receiving BT actuations. We have previously adopted computational fluid dynamics to show that the observed airway dilation is predicted to lower resistance at the airway level [11]. This is consistent with the reduction in plethysmographically determined airway resistance and increase in FEV_1_ observed in the present study 6 months after BT. Further, we here also report a significant reduction in RV after BT. Together these findings suggest that the dilatation observed in the proximal airways may have effects that are translated downstream to smaller airways, causing them to reopen, and thus reducing gas trapping. If this is true, then improvements in ventilation homogeneity would be expected—and indeed, that has been demonstrated here using MBNW. Supporting this concept, the improvements in airway resistance demonstrated in this study are significantly correlated with the improvements in LCI. Whilst it might appear that the change in MBNW after BT is small, it must be remembered that BT treatment is delivered to only a fraction of the available airways. Since there is no dilation in untreated airways on CT, the change in LCI must reflect an averaging of a larger change in treated airways with no change in untreated airways.

As a test that could be used in widespread clinical practice to assess the effects of BT, we found MBNW difficult to perform in these severely obstructed patients. It often took 10–15 min before nitrogen concentration in the expired air had washed out to less than 2%, and some patients found the dry air unpleasant and difficult to inhale over many minutes. This may have introduced some increased biological variation in the signal, and we think it is likely to explain why the trend towards improvement in Sacin was not statistically significant. Difficulty in obtaining reliable MBNW measurements has been recognized previously in patients with severe airflow obstruction [28, 29]. By comparison, we felt that the plethysmographic measurements were easier to obtain and more reliably performed by patients, and therefore a better tool to use in the routine physiological assessment of response to BT.

## Conclusion

Clinical improvement after BT is accompanied by clear improvements in lung physiology. The present study demonstrates normalisation of ventilation homogeneity after BT that can be explained by airway dilation, reduced resistance and gas trapping.

## Data Availability

The datasets used during the current study are available from the corresponding author on reasonable request.

## Abbreviations

BT: Bronchial thermoplasty
CT: Computerized tomography
MRI: Magnetic resonance imaging
MBNW: Multiple breath nitrogen washout
ERS/ATS: European Respiratory Society/American Thoracic Society
ACQ: Asthma Control Questionnaire (5-item version)
ANOVA: Analysis of variance
FRC: Functional residual capacity
TLC: Total lung capacity
FEV1: Forced expiratory volume in 1 s
VC: Vital capacity
RV: Residual volume
Raw: Total airway resistance
sRaw: Specific airway resistance
LCI: Lung clearance index
Sacin: Phase III acinar slope
Scond: Phase III conductive slope
